# A Label-Free Aptasensor for Turn-On Fluorescent Detection of Aflatoxin B1 Based on an Aggregation-Induced-Emission-Active Probe and Single-Walled Carbon Nanohorns

**DOI:** 10.3390/foods12234332

**Published:** 2023-12-01

**Authors:** Huanhuan Yang, Lei Lv, Mengyu Niu, Dongjie Zhang, Zhijun Guo

**Affiliations:** 1 College of Food Science, Heilongjiang Bayi Agricultural University, Daqing 163000, China; yanghuanhuan@ccsfu.edu.cn; 2 College of Life Science, Changchun Normal University, Changchun 130032, China; 3 College of Agriculture, Yanbian University, Yanji 133002, China; lvlei@ybu.edu.cn (L.L.); myniuybu@163.com (M.N.)

**Keywords:** aflatoxin B1, aptasensor, aggregation-induced emission, single-walled carbon nanohorns

## Abstract

The determination of the aflatoxin B1 (AFB1) content has received widespread attention in the context of food safety, which is a global public health issue. Accordingly, a label-free and turn-on fluorescent AFB1 determination method is developed herein with an ss-DNA aptamer as the recognition element, 4, 4-(1E,1E)-2, 2-(anthracene-9, 10-diyl) bis(ethene-2, 1-diyl) bis(N, N, N-trimethylbenzenaminium iodide) (DSAI) as the aggregation-induced emission (AIE) fluorescent probe, and single-walled carbon nanohorns (SWCNHs) as the selective part with a fluorescence quenching effect. In the presence of AFB1, the AFB1-specific aptamer undergoes a structural transformation and switches from being a random helix to a folded structure. DSAI’s fluorescence is protected as a result of the resistance of the transformed aptamer adsorbed on the SWCNHs’ surface. Because DSAI’s fluorescence is not quenchable, the fluorescence intensity is calculated as a function of the AFB1 concentration. By simply mixing DSAI, aptamer, AFB1 samples, and SWCNHs, the method can be carried out in 2 h, with a limit of detection (LOD) of 1.83 ng/mL. It has a high selectivity in the presence of other mycotoxins, and its performance is confirmed in soybean sauce with a known concentration of AFB1. The LOD was 1.92 ng/mL in the soy sauce samples and the recovery ranged from 95 to 106%, implying that the presented aptasensor has great potential for food analysis.

## 1. Introduction

Aflatoxins are generated by *Aspergillus parasiticus* and *Aspergillus flavus,* are typically considered carcinogenic and toxic secondary metabolites, and are the most prevalent and toxic of all mycotoxins [1,2]. They are frequently present in agricultural and food products and result in serious financial and health issues for many nations [3,4]. Among the extremely toxic varieties of aflatoxins, aflatoxin B1 (AFB1) is the most toxic, followed by aflatoxin B2 (AFB2), aflatoxin G1 (AFG1), and aflatoxin G2 (AFG2) [5]. AFB1 not only contaminates cereals, grains, milk, maize, peanuts, and animal feed, but also has pathogenicity and carcinogenicity for humans and animals [6,7]. AFB1 is a category 1 carcinogen in the categorization given by the International Agency for Research in Cancer [8]. Because of growing concerns about food safety and economic factors, many countries have developed regulations with regard to the maximum permissible level of AFB1 in foods and in animal feed. For instance, the European Union limits the amount of AFB1 in grains, dried fruits, and ground nuts to 2 mg/kg [9]. The upper limit for the amount of aflatoxins in food in South Korea is 10 mg/kg [10]. Given its frequent occurrence and high toxicity, sensitive, focused, and quick analytical techniques are required for quantifying AFB1 at the trace level. The traditional techniques currently used for AFB1 detection include thin-layer chromatography (TLC) [11], liquid chromatography–mass spectrometry (LCS) [12], and high-performance liquid chromatography (HPLC) [13]. These methods, however, are time-consuming and call for difficult pretreatment procedures, specialized and expensive equipment, and trained operational personnel [14]. These drawbacks prevent these techniques from being widely used and prevent them from meeting the demands of quick and inexpensive screening analyses. Enzyme-linked immunosorbent assay (ELISA) [15], lateral flow immunoassay (LFIA) [16], fluorescence polarization immunoassay (FPIA) [17], and additional analytical techniques incorporating immunological analysis have all been used for the quick identification of AFB1 [18]. Although they work well, these analytical techniques, which are similar to the traditional techniques, have several drawbacks. Their high cost, instability, and unsuitability for preservation together render them unsuitable for meeting the current requirements for use as antibody-based detection methods.

Aptamers are single-stranded DNA or RNA oligonucleotides that have the capacity to attach to specific targets. The selection process of aptamers is referred to as SELEX (Systematic Evolution of Ligands by Exponential Enrichment) [19]. They exhibit special qualities and, hence, have drawn interest from many different disciplines. They are considered possible substitutes for antibodies, in particular, because of their capacity to fold into particular secondary and tertiary folded structures, like stem rings, hairpins, and G-quadruplexes [20,21]. Additionally, they have acquired the ability to attach to ligands with a high affinity and specificity on account of their distinct spatial structures. Aptamers are therefore used to identify a vast number of targets, including small molecules, nucleotides, proteins, cells, and organisms. They have been used extensively in bioanalytical applications as a suitable substitute for antibodies on account of their convenient methods of synthesis, maintenance, and delivery, as well as their comparable detection affinities.

Nanomaterials have been utilized more and more to build analytical devices that are target-sensitive, highly selective, and economically advantageous [22]. The development of novel analytical instruments frequently makes use of nanomaterials like nanohorns [23], nanotubes [24], nanoparticles [25], nanorods [26], and nanosheets [27]. Particularly because of their distinctive chemical, optical, mechanical, and electrical characteristics, carbon nanomaterials are attracting interest on a global scale [28]. As a new kind of carbonaceous nanostructured material, single-walled carbon nanohorns (SWCNHs) are horn-shaped sheaths comprising single-walled graphene sheets. Many unique properties result from the individual SWCNHs’ horn-shaped structure and the aggregated SWCNHs’ spherical structure [29]. SWCNHs have been widely utilized in a series of applications, such as adsorption, drug delivery, and bio-sensing.

In recent years, many new and original aptasensors for AFB1 determination have been developed. These were mainly constructed based on fluorescence [30,31,32], electrochemical transducers [33,34,35], and colorimetric transducers [36,37,38,39]. In particular, for electrochemical transducers, electrode processing is problematic and tedious; for colorimetric transducers, the sample color can affect the result. In the development of new aptasensors, fluorescent aptasensors are one of the most explored research domains. Their advantages include simplicity and convenience of detection, high sensitivity, and a capacity for high-throughput detection. For example, Chen and co-workers developed a novel aptasensor employing a carboxyfluorescein (FAM)-modified aptamer and a partially complementary DNA modified with a carboxytetramethylrhodamine (TAMRA) quenching group to detect AFB1 [40]. Joo and co-workers developed a rapid assay using a fluorescein amidite (FAM)-labeled aptamer and graphene oxide (GO) as a fluorescence quenching element to detect AFB1 [41]. However, in these assays for AFB1 detection, the aptamer needs to be modified. DNA modification is a time-consuming and expensive procedure, and most of the time, aptamers adhere to the fluorophore labels. Furthermore, the aptamers’ binding properties are presumably changed as a result of the type of label and/or quenching molecules used, which could compromise test results. In addition, advancements in fluorescence approaches have thus far been constrained by the shortcomings of the labeled dyes (for instance, near-infrared dyes) that are commonly employed in tests, such as low emission intensity and poor photostability [42]. A new label-free method for AFB1 detection has thus become essential.

Fluorophores that demonstrate aggregate-induced emission (AIE) are nonemissive in the molecularly dissolved state but emit intensely bright light when in the aggregate state [43]. AIE molecules’ intramolecular rotations are capable of deactivating the corresponding excited states, rendering them nonemissive in the corresponding solutions, as demonstrated by experimental and theoretical research. In the aggregated forms, the steric interactions between molecules are blocked, and as a result, their emissions are increased.

In the current research, we used SWCNHs and 4, 4-(1E,1E)-2, 2-(anthracene-9, 10-diyl) bis(ethene-2, 1-diyl) bis(N, N, N-trimethylbenzenaminium iodide) (DSAI) to develop a novel label-free fluorescence aptasensor for AFB1 determination. In order to achieve turn-on and label-free AFB1 determination, DSAI was employed as a water-soluble AIE fluorescent probe. This probe has a turn-on fluorescence characteristic when it aggregates on bio-molecular surfaces, but it lacks selectivity for any particular biomolecule. These characteristics led to the use of DSAI as the fluorescent dye in the suggested detection device. This method has many advantages, such as its high sensitivity, ease of operation, high specificity, and significantly lower cost, without needing to modify the aptamer.

## 2. Experimental Section

### 2.1. Materials 

The AFB1 aptamer (5′-GTT GGG CAC GTG TTG TCT CTC TGT GTC TCG TGC CCT TCG CTA GGC CCA CA-3′) [42] was procured from Shanghai Sangon Biotechnology Co., Ltd. (Shanghai, China). A 10 mM Tris buffer (pH 8.0, 120 mM NaCl, 5 mM KCl, 10 mM MgCl_2_, and 20 mM CaCl_2_) was utilized to prepare the DNA storage solution and was maintained at −20 °C. The SWCNHs were procured from Nanjing Jicang Nanotechnology Co., Ltd. (Nanjing, China). The DSAI was generously donated by Professor Wenjing Tian of Jilin University. The mycotoxins AFB1, AFB2, aflatoxin M1 (AFM1), ochratoxin A (OTA), Zearalenone (ZEA), and fumonisin B1 (FB1) were procured from Sigma-Aldrich (St. Louis, MO, USA). The soybean sauce was procured from North Kang Brewing Food Co., Ltd. (Changchun, China). The other compounds were all of analytical grade and were utilized directly without further purification. Deionized (DI) water obtained using Milli-Q ultra-high-purity water equipment (Millipore, Bedford, MA, USA) was employed in the preparation of all solutions. 

### 2.2. Instrumentation

An RF-5301PC fluorescence spectrophotometer (Shimadzu, Tokyo, Japan) with a 150 W Xenon lamp as the excitation source was utilized to record the fluorescence spectra. The emission spectra were measured between 460 and 650 nm upon 430 nm excitation. The excitation and emission slits were all set at 5 nm. A Cary 500 Scan UV–VIS spectrophotometer (Varian, USA) was utilized to quantify the oligonucleotides. Transmission electron microscopy (TEM) was implemented using a Hitachi H600 transmission electron microscope (Hitachi, Tokyo, Japan) operated at an accelerating voltage of 100 KV. Scanning electron microscopy (SEM) was implemented using a Hitachi SU8010 scanning electron microscope (Hitachi, Tokyo, Japan) operated at an accelerating voltage of 5 KV. The HPLC chromatograms were captured using an Agilent 1260 HPLC system (Agilent Technologies, Palo Alto, CA, USA) with fluorescence detectors. All measurements were carried out at room temperature.

### 2.3. Fluorescent Detection of AFB1

The aptamer was heated to 95 °C for 5 min prior to the experiments, and it was then cooled to room temperature. For each examined concentration of AFB1, a mixture of 100 μL of 200 nM aptamer solution (final concentration 50 nM) and 100 μL of solution containing AFB1 was allowed to incubate for 30 min at room temperature. Subsequently, 100 μL of 12 μM DSAI solution (final concentration 3 μM) was added to the mixture, which was then incubated for 30 min at room temperature in the dark. Finally, 100 μL of 20 μg/mL SWCNH solution (final concentration 5 μg/mL) was added, bringing the mixture’s total volume to 400 μL. The mixture was incubated for 30 min at room temperature in the dark, and its fluorescence intensity was then measured.

### 2.4. Application 

Soybean sauce samples spiked with various concentrations of AFB1 (10, 20, 50, 100, and 200 ng/mL) were subjected to test trials to see how well the suggested sensor system performed when analyzing real samples. By examining the fluorescence intensities, the AFB1 concentrations in the samples were ascertained.

### 2.5. Sample Preparation for HPLC

A volume of 2 mL of methanol/water (80/20, *v*/*v*) extraction solution was utilized to ultrasonically extract 8 mL of soybean sauce for 20 min. After filtration through filter paper, 2 mL of the filtrate was passed through an immunoaffinity column at a flow velocity of approximately 1 drop per second. The immunoaffinity column was washed with 2 mL of water at a flow velocity of 1–2 drops per second until 2–3 mL of air had passed through the column. Then, 1 mL of methanol was utilized to elute the analytes into a vial at a flow velocity of 1 drop per second. The eluates were evaporated to dryness under a stream of nitrogen at 45 °C. Prior to HPLC analysis, 500 µL of methanol was employed to redissolve the residues, which were then filtered through 0.22 µm filters.

### 2.6. HPLC-FLD Conditions

A C18 column (Agilent SB-C18, 150 mm × 4.6 mm, 5 μm) was utilized to perform HPLC analysis in an Agilent 1260 HPLC chromatographic system under isocratic conditions with the column temperature maintained at 30 °C. Water/methanol/acetonitrile (42:40:18, *v*/*v*) was used as the mobile phase. The sample (10 μL) was injected at a flow velocity of 1 mL/min. FLD was obtained by means of a Agilent 1260 G1321C Fluorescence Detector (λ_ex_ = 360 nm, λ_em_ = 450 nm). 

### 2.7. Statistical Analysis

All presented data are averages of at least three independent measurements, with an experimental error of 10% or less. Origin 8.5 software was employed to process and analyze the initial data.

## 3. Results and Discussion

### 3.1. Analytical Principle of AFB1 Determination 

**Scheme 1:**
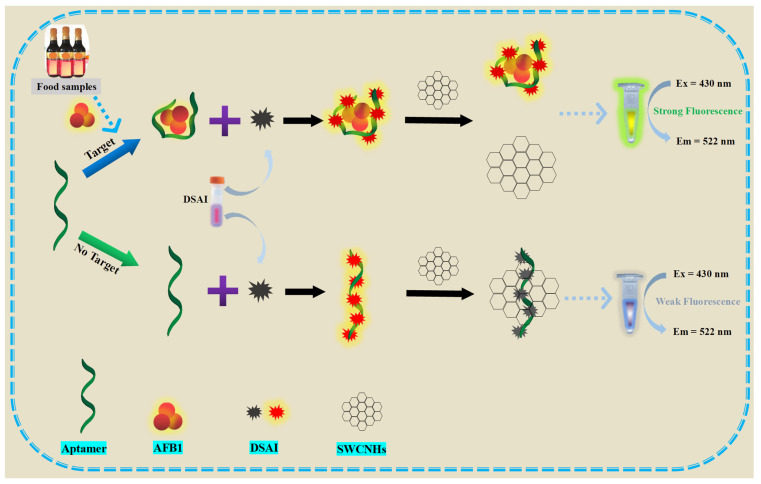
Schematic representation of the operation of the label-free aptasensor for AFB1 determination.

The aptasensor’s foundation lies in the three components’ functions: DSAI’s role as a fluorescent probe, the target-induced conformation of the folded aptamer structures, and the adsorption of unfolded ssDNA by SWCNHs. The electrostatic forces between the phosphate anion backbone within the aptamer and the ammonium cation in DSAI, as well as the hydrophobic interactions between the nucleosides in the aptamer and the aryl rings in DSAI, caused the formation of an aggregation complex of DSAI and aptamer (DSAI/aptamer complex) upon the addition of DSAI [44]. As a consequence, it would be reasonable to anticipate that the fluorescence of the complex would be improved due to DSAI’s intramolecular rotation being restricted in the agglomerated state, which closes the nonradiative decay channel. SWCNHs are π-rich nanocarbons. Therefore, there should be a powerful “π−π” interaction between the nucleotide ring structures in the nucleotide bases and SWCNHs’ hexagonal cells [45]. As a consequence of ssDNA adhering to the surface of SWCNHs, the fluorophore DSAI comes into contact with SWCNHs, quenching the fluorescence. Scheme 1 depicts the basic principle of the developed aptasensor for AFB1 determination. In the absence of AFB1, the SWCNHs’ surface can absorb the DSAI-bound aptamer (ssDNA) due to π–−π stacking interactions. Thus, the fluorescence resonance energy transfer (FRET) from the dye to SWCNHs caused by the closeness of the fluorophore and quencher causes the quenching of DSAI’s fluorescence. On the contrary, in the presence of AFB1, the AFB1 aptamer undergoes a structural transformation and switches from being a random helix to being a folded structure. DSAI’s fluorescence is protected as a result of the resistance of the transformed aptamer adsorbed upon the SWCNH surface. Because DSAI’s fluorescence is not quenchable, the fluorescence intensity is calculated as a function of the AFB1 concentration.

### 3.2. Method Feasibility for AFB1 Determination

**Figure 1:**
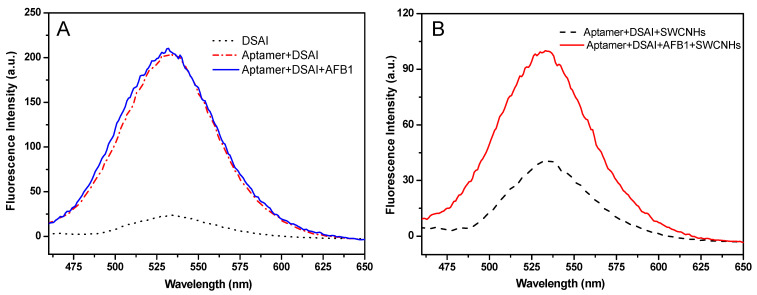
The feasibility of the AIE-active probe-based assay. (**A**) Without SWCNHs; (**B**) With SWCNHs; (final concentrations: aptamer, 50 nM; DSAI, 3 μM; AFB1, 1000 ng/mL; SWCNHs, 5 μg/mL).

By measuring the DSAI’s fluorescence intensity under different conditions, the method’s feasibility was confirmed. Figure 1 presents the corresponding fluorescence spectra. As shown in Figure 1A, DSAI is nonemissive in the molecularly dissolved state and its fluorescence was weak in solution. Upon addition of the ssDNA aptamer, the aggregation complex of DSAI and ssDNA aptamer (DSAI/ssDNA aptamer complex) was formed. Accordingly, the fluorescence of the complex was greatly enhanced. Meanwhile, the absence or presence of AFB1 had no obvious influence on the fluorescence intensity when SWCNH adsorption was not involved. On the contrary, as shown in Figure 1B, when SWCNH adsorption was involved, the sample containing AFB1 presented greatly enhanced fluorescence intensity as compared to the blank sample. This is because the aptamer–AFB1 complexes’ folded structures prevented their absorption by the SWCNHs. These results suggest that the addition of AFB1 can prevent aptamer adsorption on SWCNHs and further confirm that this platform can be used for the determination of AFB1.

### 3.3. Characterization of SWCNHs

**Figure 2:**
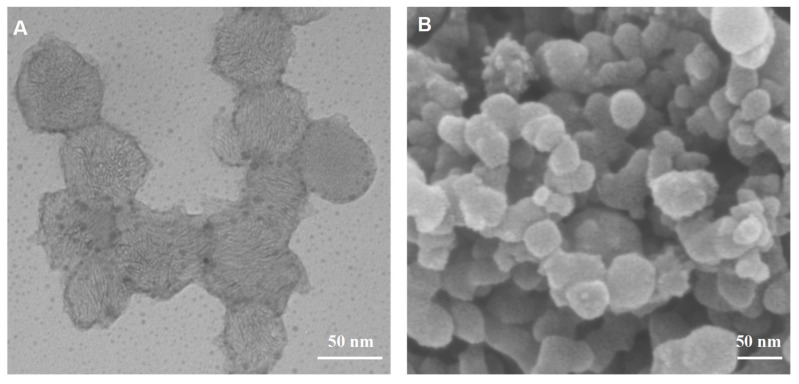
(**A**) TEM image of SWCNHs; (**B**) SEM image of SWCNHs.

Figure 2 shows TEM and SEM images of the SWCNHs. The images indicate that the SWCNH particles were spherical aggregates with diameters within the range 50–100 nm, which is in accordance with the report by Nan Li and colleagues [28].

### 3.4. Optimization of the DSAI Concentration

**Figure 3:**
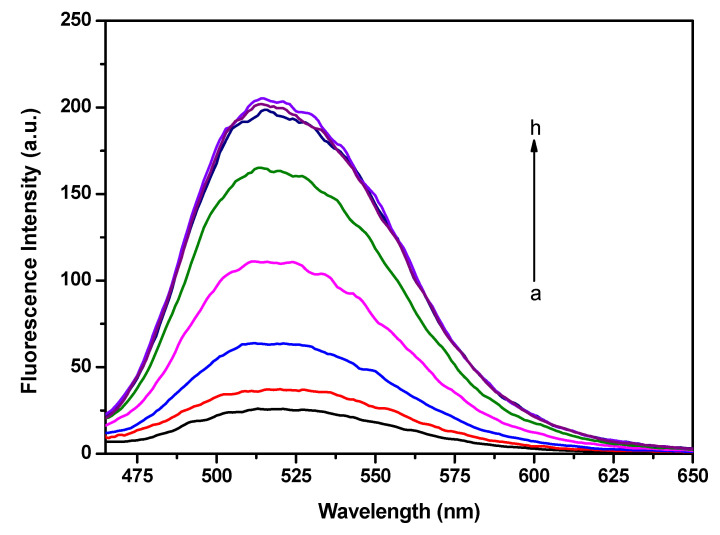
Effect of DSAI final concentration (a to h: 0.5, 1, 1.5, 2, 2.5, 3, 3.5, and 4 μM) on fluorescent enhancement. The aptamer final concentration was fixed at 50 nM.

The optimum concentration of DSAI was explored by measuring the fluorescence spectra of the aptamer reacting with different concentrations of DSAI. To pinpoint the ideal concentration of DSAI for complete reaction with the aptamer, 100 µL volumes of DSAI solutions with different concentrations were added to a fixed final concentration of DNA aptamer (50 nM) in binding buffer (to a total final volume of 400 μL). Firstly, 100 µL of 200 nM aptamer was reacted with 100 µL of different concentrations of DSAI (2, 4, 6, 8, 10, 12, 14, and 16 µM). Then, 200 μL of buffer solution was added to each of the mixtures, to a total final volume of 400 μL. The mixture was incubated for 30 min at room temperature in the dark, and its fluorescence intensity was then measured. Figure 3 displays the fluorescence spectra for different final DSAI concentrations. It shows that with increasing DSAI concentration, the maximum fluorescence intensity of the fluorescence spectrum also rose. There was no discernible rise in fluorescence intensity beyond a DSAI concentration of 12 μM (final concentration 3 μM). Therefore, a final concentration of 3 μM of DSAI was utilized in the experiments because a higher DSAI concentration would only increase the costs associated with the test.

### 3.5. Optimization of the SWCNH Concentration

**Figure 4:**
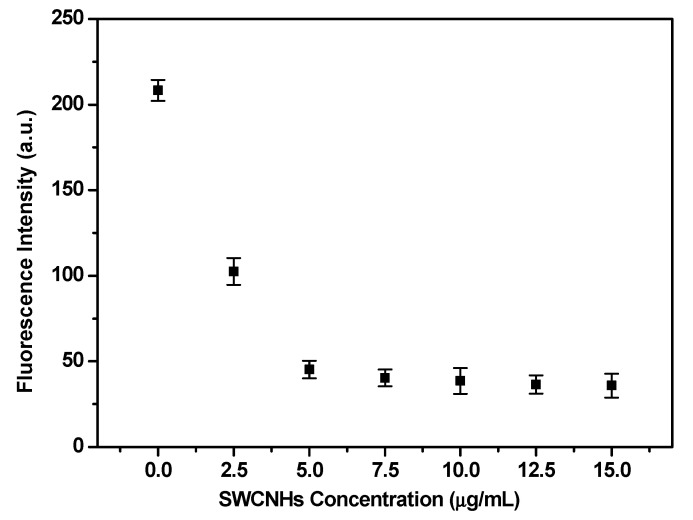
Influence of the final concentration of SWCNHs on the fluorescence intensity of the DSAI-bound aptamer (error bars were obtained from three parallel experiments).

Folded structures do not easily adsorb onto SWCNHs, even though ssDNA can be strongly adsorbed onto them. If the SWCNH concentration is too low or too high, the sensitivity will be affected. Hence, the sensitivity of the method greatly depends on the SWCNH concentration. We investigated the effects of the SWCNH concentration on the fluorescence intensity to determine the optimal SWCNH concentration for this method. Firstly, 100 µL of aptamer solution (200 nM) was mixed with 100 µL of DSAI solution (12 μM) and incubated for 30 min at room temperature in the dark. Then, 100 µL solutions of various concentrations of SWCNHs (0, 10, 20, 30, 40, 50, and 60 μg/mL) were each added to an aptamer/DSAI mixture. Finally, 100 μL of buffer solution was added to each mixture, to a total final volume of 400 μL. The mixture was incubated for 30 min at room temperature in the dark, and its fluorescence intensity was then measured. Figure 4 displays the fluorescence intensity at different SWCNH concentrations. With an increasing concentration of added SWCNHs, the maximum fluorescence intensity decreased. When the SWCNH concentration reached 20 μg/mL (final concentration 5 μg/mL), approximately 90% of the fluorescence intensity was quenched. As the concentration of SWCNHs further increased, no obvious effect was observed on the fluorescence intensity quenching. The ideal SWCNH final concentration was therefore selected as 5 μg/mL; this concentration was used in the subsequent experiments as an excess of SWCNHs could potentially lower the sensor’s sensitivity.

### 3.6. Sensitivity of AFB1 Detection

**Figure 5:**
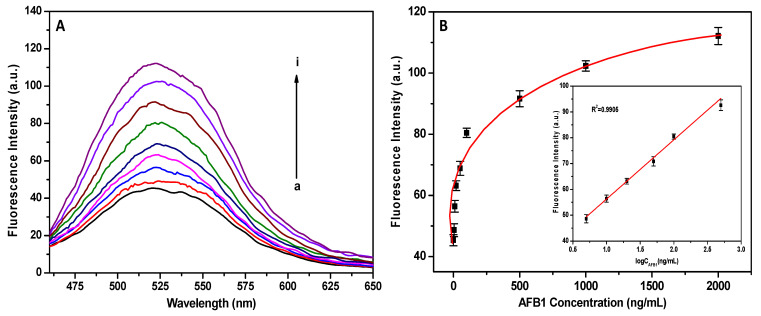
(**A**) Fluorescence spectra corresponding to different concentrations of AFB1 (0, 5, 10, 20, 50, 100, 500, 1000, and 2000 ng/mL). The excitation and emission wavelengths are 430 and 522 nm, respectively. (**B**) The highest fluorescence intensity as a function of the AFB1 concentration. Inset in B: Linear part of the plot in B. Error bars were obtained from three parallel experiments.

To consider the sensitivity of AFB1 detection, different AFB1 concentrations (0–2,000 ng/mL) were investigated. The correlation between the AFB1 concentration and the fluorescence intensity, as shown in Figure 5A, suggested that a “turn-on” aptasensor was effectively built. By conducting a linear regression analysis, the formula Y = 33.2963X + 22.9573 (Figure 5B) was derived, where X denotes the logarithm of the AFB1 concentration and Y denotes the fluorescence extent. In the concentration range from 5 to 500 ng/mL, a strong linear relationship between the parameters was discovered (R^2^ = 0.9905). The limit of detection (LOD) is described as the concentration that corresponds to the fluorescence signal at three times the standard deviation of the blank without AFB1. The LOD in this experiment turned out to be 1.83 ng/mL. Table 1 compares the performance of the current method with that of other published methods for detecting aflatoxin B1. Although the LODs for the SERS and electrochemical assays are very low [36,37], the measurement procedure is very time consuming. Similarly, for ELISA, which also has a very low LOD, the process’s antibodies have strict conservation requirements and are more likely to denature, which could ultimately have an impact on the outcomes of experiments [38]. The HPLC assay is quite durable [39]. However, it is lengthy, requires intricate pretreatment procedures, and requires specialist operational staff. The colorimetric test is straightforward to use, but it has a weak level of interference resistance [40]. The assay exhibits a “turn-off” fluorescence characteristic linked with the methodology employed [41], as an environmental stimulus or any other agent may lead to a reduction in fluorescence intensity (quenching) and subsequently produce “false positive” findings, thereby compromising the specificity of the fluorescent assay. In contrast, our method has a “turn-on” fluorescence characteristic, and its LOD is acceptable.

### 3.7. Selectivity of AFB1 Detection

**Figure 6:**
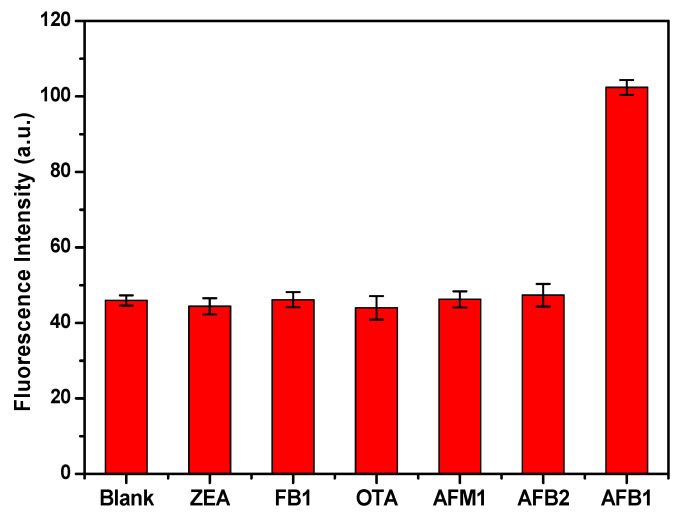
The selectivity of the method (error bars were obtained from three parallel experiments).

Selectivity is another crucial parameter of a sensor device. Test trials were conducted by making use of different structural analogues (AFB2 and AFM1) and other mycotoxins (ZEA, FB1, and OTA) to verify the method’s specificity. AFB1, AFB2, AFM1, and other mycotoxins were used in the selectivity experiment at a concentration of 1 µg/mL. The experimental findings represented in Figure 6 demonstrate that, in comparison with what was observed for the blank groups, the inclusion of the analogs and other mycotoxins did not affect the fluorescence intensity. However, the fluorescence intensity considerably increased when AFB1 was present. The column diagram effectively illustrates this variation. These results suggest a high specificity for the proposed AFB1 sensing system.

### 3.8. Analysis of AFB_1_ in Samples

An experiment was designed to confirm the practicability of the method by attempting AFB1 detection in soybean sauce. Prior to adding various AFB1 concentrations to buffer solutions containing 2% soybean sauce, HPLC was used to confirm that AFB1 was not present in the soybean sauce samples. Figure S1 presents the HPLC chromatogram of the soybean sauce, indicating that the commercial soybean sauce was AFB1-free. The LOD for the aptasensor was 1.92 g/mL for the soybean sauce samples (Figure S2). The findings of the detection experiment, which involved the addition of various known AFB1 concentrations (10, 20, 50, 100, and 200 ng/mL), are displayed in Table 2. In order to further verify the proposed assay’s applicability in real samples, HPLC was utilized to validate the present methodology. The results obtained were satisfactorily similar to the amounts of AFB1 added into the samples. The recovery rates ranged from 91.3% to 109.6%, and the relative standard derivations ranged from 2.9% to 5.6%. The results were more or less similar to those obtained via the HPLC method. These findings indicate that the suggested assay is reliable for AFB1 determination in real samples; therefore, it can be applied for the detection of other harmful substances in agricultural products. The suggested approach could be utilized to enhance and govern product security.

## 4. Conclusions

In summary, a label-free, straightforward, and sensitive aptasensor based on DSAI and SWCNHs was developed for the detection of AFB1. The proposed method possesses advantages in terms of its general applicability and simplicity. Under optimal conditions, the fluorescence intensity increased linearly with the logarithm of the AFB1 concentration in the range from 5 to 500 ng/mL, and a LOD of 1.83 ng/mL was obtained. This aptasensor also showed a high selectivity for AFB1 against its structural analogues and other mycotoxins. Satisfactory recovery rates were obtained in the measurement of AFB1-spiked soybean sauce samples. Thus, the method suggested in this research is a cost-effective, easy, and practical method for detecting AFB1. Additionally, this method exhibits excellent potential for utilization as a routine test for AFB1 contamination in a vast variety of food sources, such as dried fruits, coffee, and cereals, due to its high selectivity, simplicity, swiftness, and ability to operate at room temperature. The current work was based on the integration of specific recognition molecules (like nucleic acids) within nanostructures (like SWCNHs) for detecting and analyzing samples. This novel strategy is expected to provide a new way to develop sensitive analytical methods for the determination of various targets and to be widely used in the field of food safety.

## Figures and Tables

**Table 1:** Performance comparison of this method with other reported methods for AFB1 determination.

Method Applied	Linear Range	LOD	Reference
SERS method	1–1000 pg/mL	0.48 pg/mL	[46]
Electrochemical method	0.05–20 ng/mL	0.016 ng/mL	[35]
Immunochromatographic method	/	0.25 ng/mL	[47]
HPLC method	10–80 ng/mL	0.035 ng/mL	[48]
Colorimetric method	25–84.38 ng/mL	2.19 ng/mL	[36]
Fluorescence method	4.5–300 ng/mL	4.5 ng/mL	[41]
Fluorescence method	5–500 ng/mL	1.83 ng/mL	This work

**Table 2:** Application of the proposed assay for AFB1 detection in soybean sauce samples ^a^.

Sample Number	Added (ng/mL)	Detected (ng/mL) Mean ± SD (*n* = 3) Present Assay HPLC		RSD (%) Present Assay HPLC		Recovery (%) Present Assay HPLC	
1	10	10.9 ± 2.8	9.8 ± 1.7	3.1	2.1	109.0	98.0
2	20	18.5 ± 1.6	20.2 ± 2.4	4.6	3.2	92.5	101.0
3	50	46.9 ± 0.9	51.3 ± 1.1	2.9	1.2	93.8	102.6
4	100	109.6 ± 3.7	98.7 ± 4.2	5.6	4.9	109.6	98.7
5	200	182.6 ± 5.9	192.3 ± 3.9	3.2	3.7	91.3	96.2

^a^ The data reported in the table represent the average of three measurements.

## Data Availability

Data not available due to commercial restrictions.

## Supplementary Materials

The following supporting information can be downloaded at: https://www.mdpi.com/article/10.3390/foods12234332/s1, Figure S1: (A) Aflatoxin typical standard chromatogram with aflatoxin B1 at 50 ng/mL, B2 at 12.5 ng/mL, G1 at 50 ng/mL, and G2 at 12.5 ng/mL; (B) The HPLC chromatogram of commercial AFB1-free soybean sauce; Figure S2: The calibration plot in the presence of various concentrations of AFB1 in soybean sauce.
