# Supplementary material for: A Label-Free Aptasensor for Turn-On Fluorescent Detection of Aflatoxin B1 Based on an Aggregation-Induced-Emission-Active Probe and Single-Walled Carbon Nanohorns
Source: Foods. 2023 Dec 1;12(23):4332. doi: 10.3390/foods12234332 (PMC10705928; doi:10.3390/foods12234332)
Supplement: Supplementary file 1 [file foods-12-04332-s001.zip › foods-2572954-supplementary.pdf]

Supporting information

A label-free aptasensor for turn-on fluorescent detection of aflatoxin B1 based on an aggregation-induced-emission-active probe and single-walled carbon nanohorns

Huanhuan Yang [1,2], Lei Lv [3], Mengyu Niu [3], Dongjie Zhang [1,*] and Zhijun Guo [3,*]

[1] College of Food Science, Heilongjiang Bayi Agricultural University, Daqing 163000, China;
zhangdongjie@byau.edu.cn (D.Z.)
[2] College of Life Science, Changchun Normal University, Changchun 130032, China; yanghuanhuan@ccsfu.edu.cn (H.Y.)
[3] College of Agriculture, Yanbian University, Yanji 133002, China; lvlei@ybu.edu.cn (L.L.); myniuybu@163.com (M.N.)
* Correspondence: zhangdongjie@byau.edu.cn (D.Z.); zjguo@ybu.edu.cn (Z.G.); Tel./Fax: +86-433-2435549(Z.G.)

Sample preparation for HPLC

A volume of 2 mL of methanol/water (80/20, v/v) extraction solution was utilized to ultrasonically extract 8 mL of soybean sauce for 20 min. After filtration through a filter paper, 2 mL of the filtrate were passed through an immunoaffinity column at the flow rate of approximately 1 drop per second. Wash the immunoaffinity column with 2 mL of water at a flow rate of 1-2 drops per second until 2-3 mL of air passed through the column. Then, the analytes were eluted into the vial with 1 mL of methanol at a flow rate of 1 drop per second. The eluates were evaporated to dryness under a stream of nitrogen at 45 ℃. The residues were s redissolved in 500 μL of methanol and filtered through 0.22 μm filters prior to HPLC analysis.

HPLC-FLD conditions

HPLC analysis was performed using a C18 column (Agilent SB-C18, 150 mm×4.6 mm, 5 μm) in a Agilent 1260 HPLC chromatographic system under isocratic conditions and column temperature was maintained at 30 ℃. water/methanol/acetonitrile (42:40:18, v/v) were used as mobile phase. The sample (10 μl) was injected at a flow rate of 1 ml/min. The FLD detection was obtained by means of a Agilent 1260 G1321C Fluorescence Detector (λ_{ex} =360 nm, λ_{em} = 450 nm). **Figure S1** is the HPLC chromatogram of soybean sauce, it indicated that the commercial soybean sauce is AFB1-free.

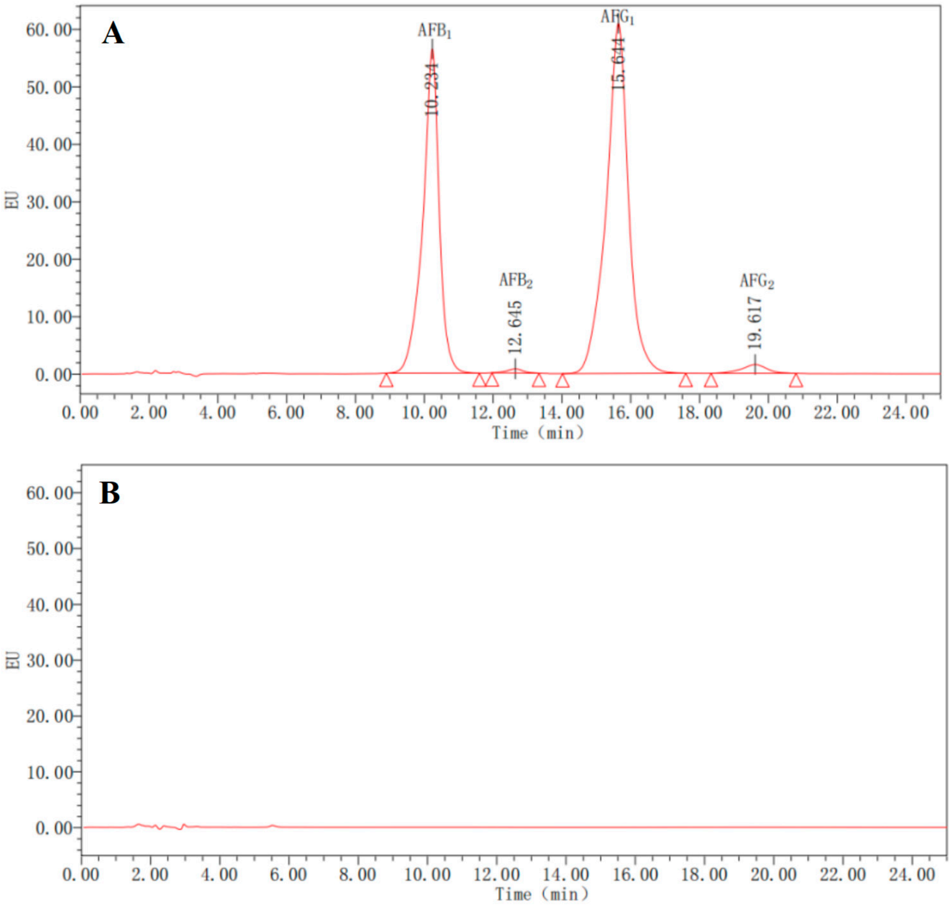

Figure S1. (A) Aflatoxin typical standard chromatogram with aflatoxin B1 at 50 ng/mL, B2 at 12.5 ng/mL, G1 at 50 ng/mL, and G2 at 12.5 ng/mL; (B) The HPLC chromatogram of commercial AFB1-free soybean sauce.

Figure S2 is the calibration plot in the presence of various concentrations of AFB1 in 2% soybean sauce samples. After conducting a linear regression analysis, the formula Y = 33.4475X + 22.5945 was derived, where the logarithm of the AFB1 concentration (X) and the fluorescence extent (Y) is represented respectively. In the concentration range from 5 to 500 ng/mL, a strong linear relationship between the parameters was discovered (R^2=0.9936). The LOD in this experiment turned out to be 1.92 ng/mL.

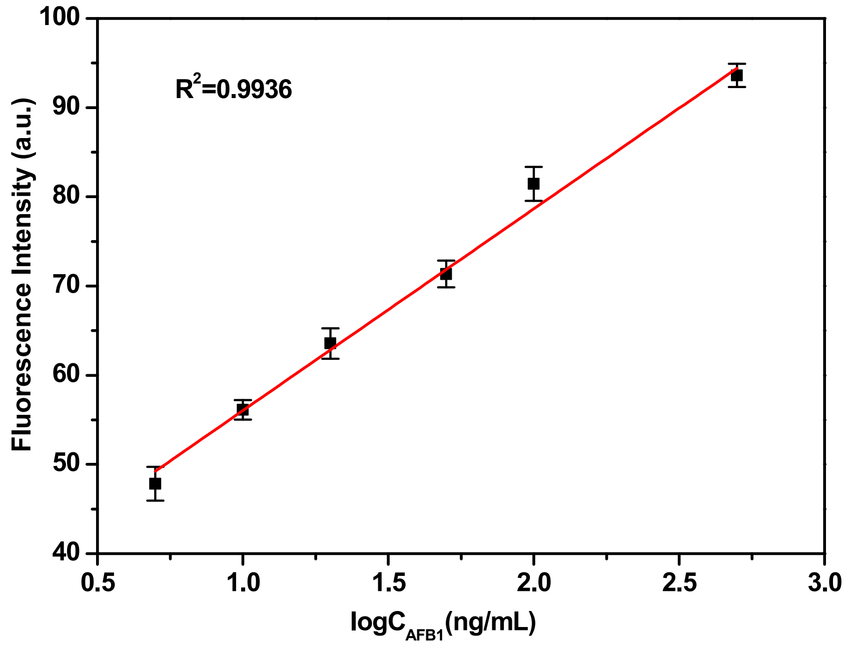

Figure S2. The calibration plot in the presence of various concentrations of AFB1 in soybean sauce.
